# Supplementary material for: Social Stigma, Mental Health, Stress, and Health-Related Quality of Life in People with Long COVID
Source: Int J Environ Res Public Health. 2023 Feb 22;20(5):3927. doi: 10.3390/ijerph20053927 (PMC10001775; doi:10.3390/ijerph20053927)
Supplement: Supplementary file 1 [file ijerph-20-03927-s001.zip › ijerph-2198399-supplementary.pdf]

Supplemental Material

Social Stigma, Mental Health, Stress, and Health-Related Quality of Life in People with Long COVID

Urte Scholz *[1 2], Walter Bierbauer [1 2], Janina Lüscher [3]

[1] Applied Social and Health Psychology, Department of Psychology, University of Zurich, Binzmuehlestrasse 14 / Box 14, 8050 Zurich, Switzerland
[2] University Research Priority Program "Dynamics of Healthy Aging", University of Zurich, Stampfenbachstrasse 73l, 8006 Zurich, Switzerland
[3] Swiss Paraplegic Research, Guido A. Zaech-Strasse 4, 6207 Nottwil, Switzerland
***** Correspondence: urte.scholz@psychologie.uzh.ch

Table S1: Correlations between main constructs and potential control variables age, and sex

Psychological measures	Age	Sex[a]
1. Total social stigma	-.07	.08
2. Enacted and perceived external stigma	-.01	.11
3. Disclosure concerns	-.04	-.003
4. Internalized stigma	-.19**	.01
5. Depressive symptoms	-.09	-.05
6. Anxiety	-.08	-.05
7. Perceived stress	-.10	.07
8. Physical hrqol	-.01	-.14*
9. Mental hrqol	.03	.02
10. Overall burden of consequences of Long COVID	-.08	-.03
11. Overall burden of Long COVID symptoms	-.13*	.07

Note. $^{*}p < .05$. $^{**}p < .01$. [a] 0 = male, 1 = female; hrqol = health-related quality of life

Table S2

Results of multiple regression analyses with enacted and perceived external stigma as predictor

	Perceived stress [a]				Depressive symptoms [b]				Anxiety [c]				Physical quality of life [d]				Mental quality of life [e]			
	beta	*b*	*b* 95% CI		*beta*	*b*	*b* 95% CI		*beta*	*b*	*b* 95% CI		*beta*	*b*	*b* 95% CI		*beta*	*b*	*b* 95% CI	
			LL	UL			LL	UL			LL	UL			LL	UL			LL	UL
Intercept		**.88****	.35	1.40		1.52	-.69	3.73		**2.76***	.44	5.08		**71.29****	63.22	79.35		**65.66****	55.98	75.34
Enacted and perceived external stigma	.25	**.17****	.08	.25	.19	**.59****	.23	.95	.09	.30	-.09	.69	-.13	**-1.02***	-1.96	-.09	-.19	**-1.52****	-2.55	-.50
Overall disease strain	.23	**.07****	.03	.11	.14	**.21***	.04	.38	-.07	-.10	-.28	.08	-.20	**-.70****	-1.14	-.26	-.17	**-.64***	-1.13	-.14
Overall symptom strain	.02	.01	-.05	.07	.04	.10	-.16	.36	.06	.14	-.14	.42	-.28	**-1.49****	-2.17	-.81	-.18	**-1.02***	-1.80	-.25
Anxiety					.46	**.45****	.34	.56												
Depressive symptoms									.49	**.50****	.38	.62								
Sex[f]													-.12	**-3.82***	-7.48	-.16				
Mental hrqol[g]													-.32	**-.30****	-.41	-.19				
Physical hrqol[g]																	-.35	**-.37****	-.51	-.24

Note. $^{*}p < .05$. $^{**}p < .01$. Empty rows indicate that the covariate was not included in the analysis. Coefficients in bold are significant. ΔR^2 is the added explained variance of total stigma predictor after controlling for covariates. [a] adjusted $R^2 = 0.12$; $\Delta R^2 = 0.06$. [b] adjusted $R^2 = 0.33$; $\Delta R^2 = 0.03$. [c] adjusted $R^2 = 0.28$; $\Delta R^2 = 0.01$. [d] adjusted $R^2 = 0.24$; $\Delta R^2 = 0.02$. [e] adjusted $R^2 = 0.16$; $\Delta R^2 = 0.03$. [f] 0 = male, 1 = female. [g] hrqol = health-related quality of life

Table S3

Results of multiple regression analyses with disclosure concerns as predictor

	Perceived stress [a]				Depressive symptoms [b]				Anxiety[c]				Physical quality of life[d]				Mental quality of life [e]			
	beta	*b*	*b* 95% CI		*beta*	*b*	*b* 95% CI		*beta*	*b*	*b* 95% CI		*beta*	*b*	*b* 95% CI		*beta*	*b*	*b* 95% CI	
			LL	UL			LL	UL			LL	UL			LL	UL			LL	UL
Intercept		**.78****	.25	1.31		1.20	-1.04	3.43		**2.50***	.24	4.76		**71.80****	63.60	80.01		**65.48****	55.83	75.12
Disclosure concerns	.19	**.12****	.04	.19	.08	.22	-.11	.55	.21	**.62****	.29	.95	-.06	-.41	-1.24	.42	-.19	**-1.43****	-2.32	-.54
Overall disease strain	.23	**.07****	.03	.11	.15	**.23***	.05	.40	-.07	-.11	-.29	.06	-.20	**-.72****	-1.17	-.28	-.16	**-.62***	-1.11	-.12
Overall symptom strain	.06	.03	-.03	.09	.08	.18	-.08	.44	.07	.15	-.11	.42	-.30	**-1.63****	-2.30	-.96	-.20	**-1.15****	-1.91	-.38
Anxiety					.47	**.46****	.35	.58												
Depressive symptoms									.47	**.48****	.36	.60								
Sex[f]													-.13	**-4.26***	-7.92	-.59				
Mental hrqol [g]													-.31	**-.29****	-.40	-.18				
Physical hrqol[g]																	-.33	**-.35****	-.486	-.22

Note. $^{*}p < .05$. $^{**}p < .01$. Empty rows indicate that the covariate was not included in the analysis. Coefficients in bold are significant. ΔR^2 is the added explained variance of total stigma predictor after controlling for covariates. [a] adjusted $R^2 = 0.11$; $\Delta R^2 = 0.04$. [b] adjusted $R^2 = 0.31$; $\Delta R^2 = 0.01$. [c] adjusted $R^2 = 0.31$; $\Delta R^2 = 0.04$. [d] adjusted $R^2 = 0.22$; $\Delta R^2 = 0.003$. [e] adjusted $R^2 = 0.16$; $\Delta R^2 = 0.04$. [f] 0 = male, 1 = female. [g] hrqol = health-related quality of life

Table S4

Results of multiple regression analyses with internalized stigma as predictor

	Perceived stress [a]				Depressive symptoms [b]				Anxiety[c]				Physical quality of life [d]				Mental quality of life [e]			
	beta	*b*	*b* 95% CI		*beta*	*b*	*b* 95% CI		*beta*	*b*	*b* 95% CI		*beta*	*b*	*b* 95% CI		*beta*	*b*	*b* 95% CI	
			LL	UL			LL	UL			LL	UL			LL	UL			LL	UL
Intercept		**.87****	.35	1.38		1.74	-.47	3.95		**3.19****	.95	5.43		**71.12****	62.86	79.39		**62.59****	53.11	72.06
Internalized stigma	.27	**.21****	.11	.30	.22	**.79****	.36	1.21	.28	**1.00****	.57	1.44	.01	.11	-.98	1.19	-.28	**-2.59****	-3.69	-1.49
Overall disease strain	.22	**.07****	.03	.11	.14	**.20***	.04	.37	-.07	-.11	-.29	.06	-.21	**-.74****	-1.19	-.30	-.14	**-.55***	-1.04	-.06
Overall symptom strain	.04	.02	-.05	.08	.06	.14	-.12	.40	.05	.11	-.15	.38	-.31	**-1.66****	-2.34	-.98	-.16	**-.93***	-1.68	-.17
Anxiety					.40	**.40****	.28	.51												
Depressive symptoms									.41	**.41****	.29	.54								
Sex[f]													-.14	**-4.31***	-7.99	-.63				
Mental hrqol [g]													-.30	**-.28****	-.39	-.16				
Physical hrqol[g]																	-.30	**-.32****	-.45	-.19

Note. $^{*}p < .05$. $^{**}p < .01$. Empty rows indicate that the covariate was not included in the analysis. Coefficients in bold are significant. ΔR^2 is the added explained variance of total stigma predictor after controlling for covariates. [a] adjusted $R^2 = 0.14$; $\Delta R^2 = 0.07$. [b] adjusted $R^2 = 0.34$; $\Delta R^2 = 0.04$. [c] adjusted $R^2 = 0.33$; $\Delta R^2 = 0.06$. [d] adjusted $R^2 = 0.22$; $\Delta R^2 = 0.00$. [e] adjusted $R^2 = 0.20$; $\Delta R^2 = 0.08$. [f] 0 = male, 1 = female. [g] hrqol = health-related quality of life

Table S5

Results of multiple regression analyses with social stigma (total score) as predictor with the subsample of participants who had an official confirmation of their Long Covid condition

	Perceived stress [a] n=190				Depressive symptoms[b] n=190				Anxiety[c] n=190				Physical quality of life[d] n=185				Mental quality of life[e] n=188			
	beta	*b*	*b* 95% CI		*beta*	*b*	*b* 95% CI		*beta*	*b*	*b* 95% CI		*beta*	*b*	*b* 95% CI		*beta*	*b*	*b* 95% CI	
			LL	UL			LL	UL			LL	UL			LL	UL			LL	UL
Intercept		**.86****	.03	1.21		1.81	-.79	4.41		1.15	-1.58	3.87		**70.06****	59.70	80.42		**67.44****	56.47	78.42
Stigma	.32	**.26****	.15	.36	.22	**.91****	.43	1.40	.20	**.77****	.26	1.28	-.16	-1.19	-2.46	.09	-.27	**-2.73****	-4.07	-1.38
Overall burden of disease	.22	**.07****	.03	.12	.13	.11	-.09	.31	.06	.10	-.11	.31	-.13	**-.59***	-1.13	-.06	-.15	**-.63***	-1.22	-.04
Overall burden of symptoms	-.01	-.003	-.08	-.07	.04	.13	-.18	.44	.05	.12	-.20	.44	-.25	**-1.47****	-2.30	-.64	-.17	**-1.08***	-2.02	-.14
Anxiety					.42	**.41****	.29	.54												
Depressive symptoms									.44	**.45****	.31	.58								
Sex[f]													-.11	-3.85	-8.73	1.02				
Mental hrqol[g]													-.33	**-.30****	-.42	-.17				
Physical hrqol[g]																	-.33	**-.36****	-.51	-.21

Note. $^{*}p < .05$. $^{**}p < .01$. Empty rows indicate that the covariate was not included in the analysis of this specific outcome. Coefficients in bold are significant. ΔR^2 is the added explained variance of total stigma predictor after controlling for covariates.
[a] adjusted $R^2 = 0.16$; $\Delta R^2 = 0.10$. [b] adjusted $R^2 = 0.35$; $\Delta R^2 = 0.05$. [c] adjusted $R^2 = 0.30$; $\Delta R^2 = 0.03$. [d] adjusted $R^2 = 0.18$; $\Delta R^2 = 0.02$. [e] adjusted $R^2 = 0.20$; $\Delta R^2 = 0.07$. [f] 0 = male, 1 = female. [g] hrqol = health-realted quality of life.

Table S6

Results of multiple regression analyses with enacted and perceived external stigma as predictor with the subsample of participants who had an official confirmation of their Long Covid condition

	Perceived stress [a] n=190				Depressive symptoms [b] n=190				Anxiety [c] n=190				Physical quality of life [d] n=185				Mental quality of life [e] n=188			
	beta	*b*	*b* 95% CI LL	*b* 95% CI UL	*beta*	*b*	*b* 95% CI LL	*b* 95% CI UL	*beta*	*b*	*b* 95% CI LL	*b* 95% CI UL	*beta*	*b*	*b* 95% CI LL	*b* 95% CI UL	*beta*	*b*	*b* 95% CI LL	*b* 95% CI UL
Intercept		**.88****	.25	1.51		1.79	-.83	4.41		.95	-1.83	3.73		**69.51****	59.22	79.80		**67.66****	56.51	78.80
Enacted and perceived external stigma	.26	**.17****	.08	.27	.21	**.65****	.26	1.03	.07	.21	-.21	.637	-.15	**-1.13***	-2.18	-.08	-.22	**-1.84****	-3.00	-.69
Overall disease strain	.23	**.08****	.03	.12	.07	.11	-.09	.31	.06	.11	-.11	.316	-1.6	**-.59***	-1.12	-.06	-.16	**-.66***	-1.26	-.06
Overall symptom strain	.01	.004	-.07	.08	.05	.13	-.18	.44	.07	.17	-.16	.50	-.25	**-.14****	-2.27	-.61	-.18	**-1.17***	-2.12	-.22
Anxiety					.47	**.46****	.34	.58												
Depressive symptoms									.50	**.51****	.38	.64								
Sex[f]													-.10	-3.73	-8.59	1.13				
Mental hrqol[g]													-.33	**-.29****	-.411	-1.68				
Physical hrqol[g]																	-.33	**-.37****	-.53	-.22

Note. $^{*}p < .05$. $^{**}p < .01$. Empty rows indicate that the covariate was not included in the analysis. Coefficients in bold are significant. ΔR^2 is the added explained variance of total stigma predictor after controlling for covariates. [a] adjusted $R^2 = 0.13$; $\Delta R^2 = 0.06$. [b] adjusted $R^2 = 0.34$; $\Delta R^2 = 0.04$. [c] adjusted $R^2 = 0.30$; $\Delta R^2 = 0.004$. [d] adjusted $R^2 = 0.18$; $\Delta R^2 = 0.02$. [e] adjusted $R^2 = 0.17$; $\Delta R^2 = 0.04$. [f] 0 = male, 1 = female. [g] hrqol = health-related quality of life

Table S7

Results of multiple regression analyses with disclosure concerns as predictor with the subsample of participants who had an official confirmation of their Long Covid condition

	Perceived stress [a] n=190				Depressive symptoms [b] n=190				Anxiety [c] n=190				Physical quality of life [d] n=185				Mental quality of life [e] n=188			
	beta	*b*	*b* 95% CI		*beta*	*b*	*b* 95% CI		*beta*	*b*	*b* 95% CI		*beta*	*b*	*b* 95% CI		*beta*	*b*	*b* 95% CI	
			LL	UL			LL	UL			LL	UL			LL	UL			LL	UL
Intercept		**.74***	.10	1.38		1.38	-1.29	4.05		.56	-2.12	3.25		**70.11****	59.64	80.58		**68.12****	56.82	79.41
Disclosure concerns	.22	**.13****	.05	.21	.10	.28	-.08	.64	.23	**.66****	.31	1.01	-.08	-0.56	-1.47	.35	-.16	**-1.23***	-2.23	-.23
Overall disease strain	.24	**.08****	.03	.13	.08	.13	-.07	.33	.07	.11	-.09	.32	-.17	**-0.62***	-1.16	-.09	-.17	**-.71***	-1.31	-.10
Overall symptom strain	.05	.02	-.05	.1	.09	.22	-.09	.53	.07	.17	-.14	.49	-.27	**-1.58****	-2.4	-.75	-.21	**-1.36****	-2.31	-.42
Anxiety					.48	**.46****	.33	.59												
Depressive symptoms									.45	**.46****	.33	.59								
Sex[f]													-.11	-4.17	-9.06	.72				
Mental hrqol [g]													-.32	**-.28****	-.40	-.16				
Physical hrqol[g]																	-.32	**-.35****	-.51	-.20

Note. $^{*}p < .05$. $^{**}p < .01$. Empty rows indicate that the covariate was not included in the analysis. Coefficients in bold are significant. ΔR^2 is the added explained variance of total stigma predictor after controlling for covariates. [a] adjusted $R^2 = 0.11$; $\Delta R^2 = 0.05$. [b] adjusted $R^2 = 0.31$; $\Delta R^2 = 0.01$. [c] adjusted $R^2 = 0.35$; $\Delta R^2 = 0.05$. [d] adjusted $R^2 = 0.17$; $\Delta R^2 = 0.01$. [e] adjusted $R^2 = 0.15$; $\Delta R^2 = 0.03$. [f] 0 = male, 1 = female. [g] hrqol = health-related quality of life

Table S8

Results of multiple regression analyses with internalized stigma as predictor with the subsample of participants who had an official confirmation of their Long Covid condition

	Perceived stress [a] n=189				Depressive symptoms [b] n=189				Anxiety[c] n=189				Physical quality of life [d] n=184				Mental quality of life [e] n=187			
	beta	*b*	*b* 95% CI		*beta*	*b*	*b* 95% CI		*beta*	*b*	*b* 95% CI		*beta*	*b*	*b* 95% CI		*beta*	*b*	*b* 95% CI	
			LL	UL			LL	UL			LL	UL			LL	UL			LL	UL
Intercept		**.87****	.25	1.48		1.96	-.66	4.58		1.53	-1.09	4.15		**69.37****	58.80	79.87		**64.98****	54.04	75.92
Internalized stigma	.05	**.24****	.15	.34	.25	**.86****	.38	1.33	.33	**1.17****	.71	1.62	-.01	-.10	-1.29	1.10	-.29	**-2.70****	-3.93	-1.48
Overall disease strain	.02	**.07****	.03	.12	.08	.12	-.08	.32	.07	-.11	-.09	.30	-.17	**-.63***	-1.17	-.09	-.14	**-.61***	-1.20	-.02
Overall symptom strain	.04	.01	-.07	.08	.16	.18	-.13	.48	.05	.12	-.19	.42	-.28	**-1.60****	-2.44	-.77	-.17	**-1.10***	-2.03	-.17
Anxiety					.07	**.38****	.24	.51												
Depressive symptoms									.37	**.38****	.24	.51								
Sex[f]													-.11	-4.10	-9.03	.83				
Mental hrqol [g]													-.31	**-.27****	-.39	-.14				
Physical hrqol[g]																	-.29	**-.32****	-.47	-.17

Note. $^{*}p < .05$. $^{**}p < .01$. Empty rows indicate that the covariate was not included in the analysis. Coefficients in bold are significant. ΔR^2 is the added explained variance of total stigma predictor after controlling for covariates. [a] adjusted $R^2 = 0.17$; $\Delta R^2 = 0.11$. [b] adjusted $R^2 = 0.35$; $\Delta R^2 = 0.04$. [c] adjusted $R^2 = 0.38$; $\Delta R^2 = 0.08$. [d] adjusted $R^2 = 0.16$; $\Delta R^2 = 0.00$. [e] adjusted $R^2 = 0.21$; $\Delta R^2 = 0.08$. [f] 0 = male, 1 = female. [g] hrqol = health-related quality of life .
